# Delayed Management of Concurrent Coronal Extrusions and Root Fractures in Two Traumatized Maxillary Immature Permanent Central Incisors: A Case Study

**DOI:** 10.3390/jcm14103605

**Published:** 2025-05-21

**Authors:** Thi Thuy Tien Vo, Thi Ngoc Anh Do

**Affiliations:** Faculty of Dentistry, Nguyen Tat Thanh University, Ho Chi Minh City 700000, Vietnam; vtttien@ntt.edu.vn

**Keywords:** extrusive luxation, immature root, orthodontic repositioning, root fracture, traumatic dental injuries

## Abstract

**Background:** The combination of two or more different types of traumatic dental injuries occurring concurrently to the same tooth presents a significant clinical challenge. By focusing on a rare combination of injuries, this case study explores the issues of delayed management of root fractures accompanied by coronal extrusions in immature maxillary permanent central incisors, underscoring the necessity for tailored approaches when guidelines for intervention were unmet. **Methods:** The case involves an eight-year-old boy who delayed seeking care for approximately a year after suffering trauma to his upper front teeth in a fall accident at school. The clinical examination revealed partial displacement of two maxillary central incisors in an incisal direction, resulting in increased mobility. Radiographs further showed horizontal root fractures in the apical third of both extruded incisors. Encouragingly, the injured teeth exhibited a normal response to electric pulp testing without signs or symptoms of pulpal pathology, suggesting pulp vitality and eliminating the need for root canal treatment. The extruded coronal fragments were repositioned orthodontically using a utility arch. **Results:** At the 14-month follow-up, the affected incisors were clinically asymptomatic, functionally satisfactory, and esthetically pleasing. **Conclusions:** Conservative orthodontic management of extrusive luxation concomitant with root fracture in immature permanent teeth may prove effective in select cases, particularly when long-term follow-up and proper oral care are maintained.

## 1. Introduction

Traumatic dental injuries (TDIs), which can be any type from enamel infractions to luxations and even avulsions, pose a significant health problem worldwide. They affect up to 22.7% of individuals with primary dentition and 15.2% of those with permanent dentition [1]. School-aged children are at higher risk of experiencing such injuries, with incisors, particularly the maxillary central incisors, being the most commonly affected teeth [2]. TDIs can cause not only physical but also esthetic and social compromises for patients, especially in pediatric cases, which call for prompt and proper management to improve outcomes and prevent complications.

Dealing with TDIs generally presents a challenge to practitioners. The management may be further complicated in immature teeth due to their distinct anatomical and physiological characteristics [3]. When dental trauma occurs, initial management has a decisive role in healing outcomes and, consequently, the prognosis of the injured teeth. Improper, delayed, and/or inconsistent treatment can result in far-reaching implications that, despite much effort, may be irreparable later, especially in children and adolescents [4]. In some cases, it is unfeasible or impossible for the patient to receive appropriate care in a timely manner, mainly due to diagnostic challenges, patient unawareness or non-compliance, and limited access to specialized healthcare facilities, thereby exacerbating the difficulty and complexity of the situation [5]. Notwithstanding progress in evidence-based guidelines, the delayed management of TDIs remains understudied.

Trauma to the dento-alveolar region typically results in tooth fractures and/or displacements, bone crushing and/or fracturing, and soft tissue injuries. The most common types of such injuries are crown fractures and luxations [6]. In contrast, root fractures, which involve the dentin, cementum, and pulp, are relatively rare, occurring in only 2% to 4% of primary dentition and 0.5% to 7.0% of permanent dentition [7]. These fractures are typically classified as vertical or horizontal, with the latter also occurring as oblique fractures with varying orientations. Vertical root fractures commonly necessitate tooth extraction and prosthesis replacement. In horizontal root fractures, the apical fragment generally shows no displacement, while the coronal fragment exhibits various dislocations [8]. That loosening and partial displacement of the tooth out of its socket in an axial direction, also known as extrusive luxation or simply extrusion, leads to increased tooth mobility, periodontal ligament disruption, and changes in the pulp or pulp circulation [9]. It is suggested that teeth with incomplete root development are less prone to root fractures than those fully developed, probably owing to the greater elasticity of the tooth socket in immature teeth [10]. In fact, only 13 incompletely developed teeth with root fractures in 11 patients have been documented [8]. The low number of cases demonstrates that young children whose teeth have not fully developed rarely sustain this type of injury. Due to the scarcity of the literature, case reports remain an essential source of data.

While the prevalence of injuries co-occurring with root fractures has not been reported [11], root fractures with coronal extrusions are considered rare yet clinically complex scenarios that warrant attention. The consequences can affect either the hard tissues in the form of fractures and dislocations, or the soft tissues that include the pulpal and the periapical tissues, or both. Traumatized teeth with delayed or no treatment have been shown to likely develop pulp canal obliteration, pulp necrosis, root resorption, and loss of marginal bone support [8,12,13]. A retrospective study of 400 root-fractured permanent incisors demonstrated that optimal repositioning of root fractures, even with coronal fragment displacement of up to 1 mm, may promote both pulpal healing and hard tissue repair [14]. The International Association of Dental Traumatology (IADT) guidelines have recommended a treatment approach that involves manual repositioning of the traumatized tooth, followed by passive and flexible splinting for at least 4 weeks [6]. Nevertheless, there are instances where manual repositioning may be impractical, such as when there is a considerable extrusion greater than three mm or when a blood clot forms within the socket as a result of delayed treatment. Surgical repositioning and orthodontic repositioning are two viable alternative therapeutic options to consider in such cases [15]. Today, evidence exists from few publications, with a very limited number of teeth involved, about the efficacy of three treatment approaches.

This report presents the case of an eight-year-old boy who sustained concomitant coronal extrusions and apical root fractures in two maxillary central incisors. Despite the established guidelines from the IADT recommending immediate repositioning and stabilization [6], the injuries in this case were not treated promptly. This delay raised concerns about potential complications such as bruised pulp and blood clots at the fracture site, which can impede tooth relocation and negatively affect prognosis [9]. Therefore, determining the appropriate treatment course in this case was challenging. A thorough diagnosis, clearly defined treatment objectives, and continuous monitoring were essential to ensure favorable outcomes and enable timely intervention if complications emerged. This case study explores the challenges of delayed management in the treatment of concurrent coronal extrusions and root fractures in immature maxillary permanent central incisors, with a focus on treatment approaches and long-term outcomes.

## 2. Case Presentation

### 2.1. Case History

An eight-year-old male patient presented with the chief complaint of increased mobility in his two upper central incisors. The child had sustained a traumatic injury to his upper front teeth in a fall accident at school. At that time, the two upper central incisors were slightly elongated and uncomfortable when touched. The labial gingiva surrounding both teeth was swollen. However, due to a lack of parental awareness, no treatment was initiated following the trauma until approximately one year later, when the patient presented to our clinic. The child had no previous dental history, and his past medical history was insignificant.

### 2.2. Preoperative Examination

**Figure 1:**
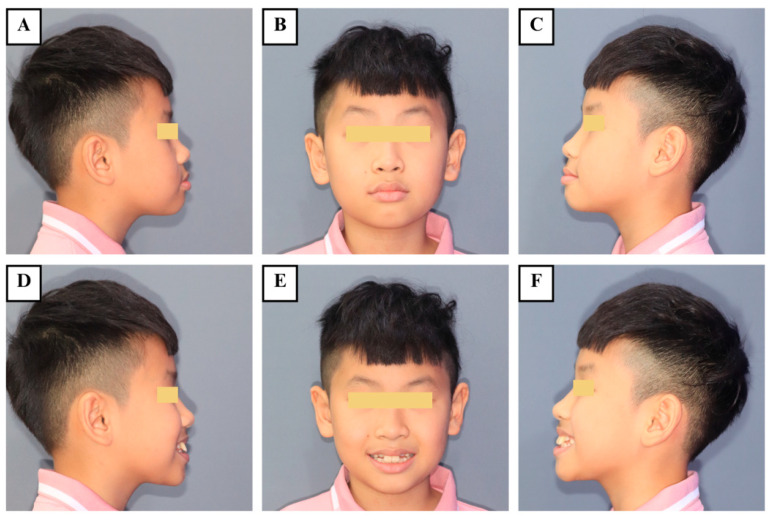
Preoperative extraoral photographs. (**A**) Right profile repose; (**B**) repose; (**C**) left profile repose; (**D**) right profile natural smile; (**E**) natural smile; and (**F**) left profile natural smile.

**Figure 2:**
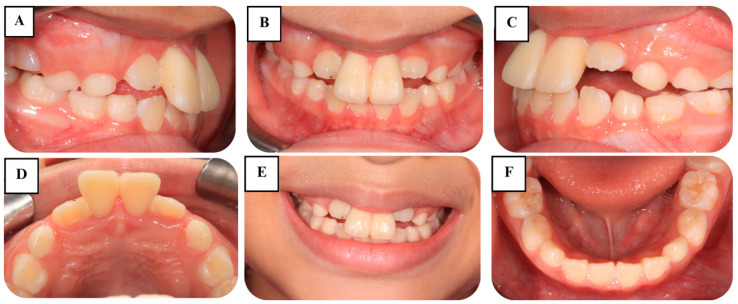
Preoperative intraoral photographs. (**A**) Right lateral view; (**B**) frontal view; (**C**) left lateral view; (**D**) maxillary occlusal view; (**E**) natural smile; and (**F**) mandibular occlusal view.

Upon clinical examination, no signs of extraoral injury were noted (Figure 1). Two maxillary central incisors (teeth #11 and #21) showed signs of mild extrusion and excessive mobility. In both teeth, the clinical severity of extrusion was 2 mm, and tooth mobility was categorized as grade III (Figure 2). Neither tenderness to percussion or palpation nor tooth discoloration was observed in the traumatized teeth. Electric pulp testing (EPT) indicated positive responses, suggesting vital pulp in the two injured incisors. There was no evidence of TDIs in other teeth or alveolar structures. Periodontal findings included reddish gingiva, moderate plaque and calculus, and bleeding on probing.

**Figure 3:**
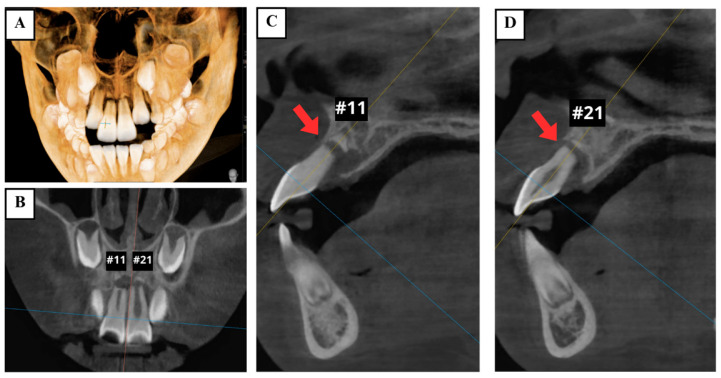
Preoperative CBCT images showing horizontal root fractures in the apical third of two maxillary central incisors (arrows). (**A**) 3D reconstruction; (**B**) coronal view; (**C**) sagittal views of the right maxillary central incisor (tooth #11); and (**D**) left maxillary central incisor (tooth #21).

Cone-beam computed tomography (CBCT) demonstrated the presence of horizontal root fractures in the apical third of both maxillary central incisors, leading to separation of the apical and coronal fragments. The traumatized teeth had incomplete root development, wide root canals, and open apices (Figure 3).

### 2.3. Diagnosis

Based on clinical and radiographic assessment, the patient was diagnosed with extrusions of the coronal fragments concurrent with horizontal root fractures in the apical third in two maxillary immature permanent central incisors. Moderate gingivitis was also noted.

### 2.4. Treatment Plan

The primary treatment objective was to preserve the two injured incisors for as long as they remained asymptomatic, while simultaneously restoring both function and esthetics. In this case, gingivitis treatment was crucial due to the possibility of serious complications, such as pulp necrosis and infection in the affected teeth, resulting from bacterial penetration [16]. According to the IADT guidelines, the first treatment option for the traumatized teeth should involve repositioning of the displaced coronal fragments as soon as possible, and stabilization of the mobile segments with a flexible splint for 4 weeks or longer [6]. However, in this scenario, where there was a significant delay in presentation, the dislocation was corrected orthodontically, considering potential healing that may have had time to occur. Furthermore, the two injured incisors showed a normal response to EPT without signs or symptoms of pulpal pathology, suggesting they did not have pulp necrosis and did not require root canal treatment. It is important to keep in mind that monitoring root-fractured teeth over time is essential to evaluate the healing response and to determine pulp vitality. If pulp necrosis develops, root canal treatment of the coronal segment to the fracture line is indicated to preserve the tooth [6,8]. Given the importance of long-term monitoring, follow-up visits were scheduled for the patient. After a thorough discussion of all treatment procedures and the prognosis of the affected teeth, written consent was obtained from the parents, and the treatment was provided on the same visit.

### 2.5. Treatment Procedures

#### 2.5.1. Phase 1: Periodontal Management

The supragingival scaling and irrigation with chlorhexidine were performed carefully to eliminate any local irritating factors that may have contributed to gingival inflammation. The patient was encouraged to practice good oral hygiene using a soft toothbrush and advised to maintain a soft diet. One week later, the gingiva showed reduced inflammation. A thorough supragingival and subgingival scaling, along with crown polishing, was then carried out. Two weeks later, the gingiva appeared normal, and the patient demonstrated proper plaque control.

#### 2.5.2. Phase 2: Orthodontic Management

During this phase, the patient was scheduled for monthly orthodontic visits. At the re-examination, clinical findings, including the position, mobility, percussion results, crown discoloration, and periodontal health of the affected teeth, were recorded. EPT was also performed to determine if changes in pulp status occur over time.

**Figure 4:**
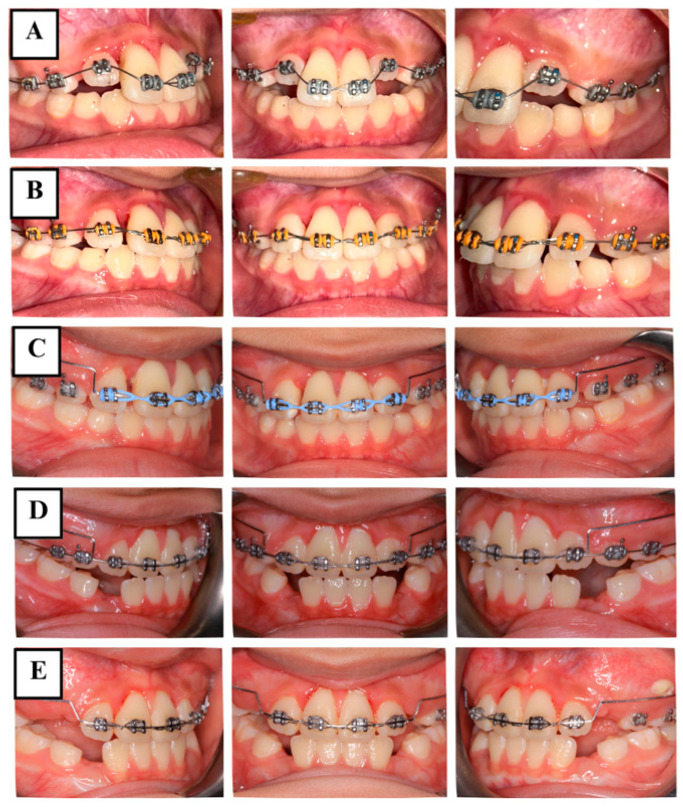
Intraoral photographs during orthodontic treatment. (**A**) Teeth alignment. (**B**) at 8 weeks. (**C**) orthodontic repositioning using the utility arch. (**D**) at 6 months. (**E**) at 14 months.

**Figure 5:**
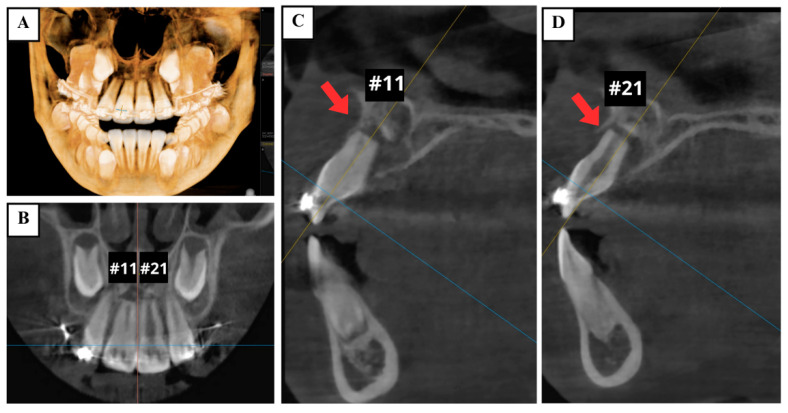
CBCT images at 14 months after orthodontic treatment showing satisfactory periodontal tissues around both the fractured fragments and the apical area (arrows). (**A**) 3D reconstruction. (**B**) Coronal view. Sagittal views of the (**C**) right maxillary central incisor (tooth #11) and (**D**) left maxillary central incisor (tooth #21).

The anterior teeth were initially aligned to allow for more effective tooth movement later (Figure 4A). Once the teeth were properly aligned after 8 weeks (Figure 4B), the coronal fragments of teeth #11 and #21 were then repositioned and stabilized using a utility arch constructed from 0.016 × 0.022-inch blue Elgilol wire (Figure 4C,D). Fourteen months later, teeth #11 and #21 remained clinically asymptomatic. The extrusion of both teeth had been corrected to 0 mm, and mobility was close to normal (Figure 4E). At this time, EPT still showed positive results. Radiographs indicated satisfactory periodontal tissues around both of the fractured fragments and the apical area, without evident radiolucency of apical inflammation or root resorption seen on the CBCT (Figure 5).

#### 2.5.3. Phase 3: Follow-Up

Routine follow-up visits should be scheduled to monitor periodontal health, pulp vitality, and the overall functional and esthetic integration of the tooth, while addressing any potential complications. According to IADT guidelines, recall appointments should be arranged annually for the first 5 years to monitor root healing, identify signs of root resorption, and detect potential complications such as apical inflammation. If no issues arise during this period, follow-up visits can be scheduled every 4 to 5 years thereafter.

## 3. Discussion

It is uncommon for a child to sustain concurrent injuries of coronal extrusion and root fracture. The involvement of both maxillary central incisors, as seen in this case, is even rarer. Despite its rarity, a root fracture is a significant injury for which practitioners should have a comprehensive understanding of different scenarios of root fractures, as well as tissue responses, management options, and prognosis of these injuries.

Various tissues are involved when an injured tooth suffers a root fracture, encompassing the root itself, the pulp, and the periodontal and periapical tissue [8]. When the majority of root fractures retain pulp vitality, the primary treatment objective is to promote the healing process. There are four types of tissue responses following root fractures, including healing with hard dental tissue, healing with connective tissue, healing with bone and connective tissue, and no healing characterized by the formation of granulation or inflammatory tissue in the fracture line due to pulp infection and pulp necrosis in the coronal fragment [7]. Teeth with root fractures generally have a good healing capacity and a favorable long-term prognosis. In a retrospective study of 400 root-fractured incisors in young patients, most of the traumatized teeth had healed by interposition of periodontal ligament alone (43%), followed by hard tissue fusion of the fragments (30%). The pre-injury or injury factors that had the greatest impact on healing included age, stage of root development, mobility and dislocation of the coronal fragment, and diastasis between fragments [17].

In extrusive luxation injury, acute treatment within three h post-trauma typically involves manual repositioning of the extruded tooth, followed by the application of a passive and flexible stabilization splint. When this approach is not achievable, the affected tooth may be repositioned surgically or orthodontically [15]. Surgical repositioning, including intentional replantation, entails extracting the extruded tooth and then immediately reinserting it into the socket. Since the procedure has several contraindications and can lead to different types of root resorption, it should be recommended only in specific situations [8,18]. Meanwhile, orthodontic repositioning results in a gentle and gradual movement of the extruded tooth back into its original position in the socket [19]. A retrospective study with five-year follow-up of extrusive luxation injuries in young patients highlighted the efficacy of orthodontic repositioning, which did not exacerbate dental pulp problems. In the study, whenever manual repositioning was not possible, orthodontic repositioning was conducted using NiTi orthodontic arch wires (0.14/0.16) inserted in preadjusted edgewise orthodontic brackets (0.018″ by 0.022″) [20]. There have also been a few reports on the favorable outcomes and high survival of extruded teeth following delayed post-traumatic orthodontic repositioning [21,22,23,24]. Although the existing literature on the use of the orthodontic repositioning approach for extrusive luxations is debatable, it still plays a role in the treatment protocols of such injuries. The lack of systematic data collection, as well as the challenges in obtaining precise and detailed superimposition of clinical data, renders it impossible to provide definitive clinical indications and, therefore, demands further research.

The present report demonstrates that two maxillary immature permanent central incisors with extruded crowns and fractured roots were successfully managed by orthodontic repositioning, without any emerging complications. This treatment approach was chosen for three primary advantages. A gentle, controlled force produced by the utility arch can mitigate the risk of additional damage to the already injured teeth. The gradual process of orthodontic repositioning may enable tissues to adapt progressively, facilitating optimal healing. The placement of orthodontic appliances is generally non-invasive and painless, thereby ensuring patient comfort [15]. Furthermore, several considerations were taken into account when determining the course of treatment for the patient.

First, dental plaque in the gingival sulcus can be a source of infection, potentially leading to inflammation if not properly controlled. Consequently, the base of the gingival crevice may shift apically, allowing the fracture line to communicate with the oral cavity, resulting in many complications and even tooth loss [25]. Therefore, periodontal management, including supragingival and subgingival scaling to remove dental plaque and calculus, as well as oral hygiene instruction to improve at-home oral care, was the initial phase of treatment to enhance the oral health of the patient.

Second, it is well-recognized that adequate management of injured soft tissue, particularly the dental pulp and, if involved, the periodontal ligament, is essential to preserve the function and longevity of the traumatized tooth [26]. The impact of each trauma type and their combined effects on overall outcomes should be considered when dealing with concomitant injuries. Displacement of the coronal fragment of a root-fractured tooth can compromise blood supply to the coronal portion, potentially leading to pulp necrosis [8]. In a large study of 400 teeth with root fractures, 22% of cases showed no healing due to pulp necrosis and inflammatory changes in the coronal fragments [17]. In contrast, the apical fragment remained unaffected, as all impact forces were absorbed at the fracture site. The literature indicates an exceptionally good prognosis for incompletely developed teeth with root fractures, with none of these teeth being lost over the 10-year follow-up period. Additionally, no cases of root canal infection, pulp necrosis, external resorption, ankylosis, or bone loss were reported [8]. Encouragingly, the two traumatized incisors in this case were immature permanent teeth, with fractures located in the apical third, which contributed to a relatively better prognosis. Due to the absence of pain and pathological signs in the coronal pulp after one year, it is likely that the damaged pulp underwent revascularization and reinnervation, eliminating the need for endodontic treatment. Our results were in accordance with previous studies that indicated more favorable outcomes in immature teeth with root fractures [14,27,28]. This is probably attributable to a larger volume of pulp tissue, greater availability of inflammatory mediators, proximity to the primary vascular supply, and a higher density of the vasculature system [10]. On the other hand, while the periodontal ligament may suffer laceration in cases of tooth luxation, regardless of tooth displacement, the overall recovery of the periodontal ligament is generally favorable [6]. In the absence of inflammatory stimuli, healing of the periodontal ligament may take approximately two weeks [29]. It has been shown that manual repositioning of luxated teeth can apply significant forces, which may further damage the periodontal ligament and neurovascular bundle. Therefore, orthodontic repositioning using light forces may be a preferable approach to manual repositioning, as it can promote better periodontal healing and reduce the risk of ankylosis [9]. However, the differences in periodontal sequelae between manual and orthodontic repositioning in extrusively luxated teeth have not yet been thoroughly investigated.

Third, the patient was not seen in our clinic until a year after the trauma. The timing of intervention in relation to the trauma, classified as acute (within 3 h), subacute (within 24 h), or delayed (after 24 h), is considered a critical determinant to the selection of treatment [15]. It has been reported that delaying treatment can lead to the formation of blood clots, which can hinder repositioning of the displaced teeth [9]. The decision not to manually reposition the traumatized incisors in this instance can be attributed to the delay in treatment, as manual repositioning could further compromise already injured teeth. In several cases, orthodontic repositioning with light, gradual, and controlled forces has proven to be a less traumatic alternative that can maintain tooth vitality, and the intervention is mostly postponed for days or weeks after the trauma [19]. A previous report described a case of complex trauma to the early mixed dentition, where tooth avulsion, intrusion, extrusion, and lateral luxation were effectively managed by using a fixed, non-rigid orthodontic splint after a traditional wire-composite splint failed. At the last follow-up visit, 19 months post-trauma, all affected teeth were clinically asymptomatic and vital, with physiological mobility. These findings suggested that orthodontic appliances may be a viable choice for managing complicated injuries [21]. Despite their efficacy, orthodontic splints should be used with caution since it is difficult to construct a passive splint that does not exert force on the teeth, thereby disrupting the healing process in traumatized teeth and leading to undesirable tooth movement [30]. In other studies, delayed multidisciplinary management of extruded permanent central incisors with necrotic pulp was reported. Upon completion of endodontic treatment, orthodontic repositioning, and esthetic restoration, the traumatized teeth were diagnosed as normal at long-term recall [22,23,24]. An illustrative example is a case that described orthodontic repositioning of an extruded maxillary right central incisor using elastic ligatures as a part of a multidisciplinary approach to delayed treatment of traumatic injuries involving extrusive luxation, avulsion, and crown fracture [24]. This technique enables simple intrusion but lacks the ability to control or guide tooth movement precisely in three dimensions. In our case, the extruded incisors exhibited grade III mobility, and orthodontic intrusion was recommended for the injured teeth because their roots were in an early stage of development with open apices, potentially favoring post-traumatic revascularization [31]. The utility arch is considered a crucial and versatile tool that can be employed at different stages of orthodontic treatment, whether in mixed or permanent dentition. With the intrusion utility arch, it is possible to control the applied force and stabilize teeth passively [32]. Our results indicate that the utility arch can effectively reposition and stabilize extruded coronal fragments of root-fractured teeth after the trauma. There is only one previous case report presenting delayed orthodontic intrusion of a traumatically extruded immature permanent incisor with a utility arch [22]. Although data are scarce, the technique can prove simple yet effective when manual repositioning of the dislocated tooth is impossible, particularly common in cases of delayed treatment. Despite some differences between studies, in all cases, orthodontic treatment consistently led to preservation of extruded teeth without emerging pathological signs, both clinically and radiographically. However, research on potential sequelae in traumatized teeth subjected to orthodontic tooth movement remains limited. Furthermore, the optimal timing for orthodontic treatment post-trauma is still controversial. Literature suggests that extrusively luxated teeth should be repositioned manually within 2 days; otherwise, orthodontic intervention is recommended [33].

Last, the success of treatment significantly depends on post-trauma care. The IADT guidelines recommend meticulous oral hygiene to maintain the oral health of a dental trauma patient [34]. In this case, both the patient and his parents were instructed on the importance of oral health to encourage proper oral hygiene practices. This would help the child establish an appropriate oral care routine, improving oral health. Additionally, timely recalls ensure adequate clinical and radiographic observations after the trauma. If signs or symptoms are noted, intervention can be implemented promptly [26]. Therefore, the patient and his parents were further informed of the importance of active follow-up visits starting from the first passive appointment, regardless of whether the problem is related to the traumatized teeth or the oral health condition. Importantly, a standardized and repeatable method of data collection, carefully carried out throughout the diagnostic and monitoring phases, may provide additional evidence that can be integrated in future studies. For this purpose, radiographic evaluation is among the most widely used modalities. With the advances in imaging, three-dimensional imaging techniques, particularly CBCT, which analyze the teeth and adjacent structures in multiple planes, have demonstrated superior accuracy in evaluation compared to conventional radiography [35].

## 4. Conclusions

Delayed treatment of dental trauma, often resulting from low public awareness, can lead to serious complications. This report highlights the critical considerations in managing traumatic injuries in pediatric patients, especially those with immature roots. Despite the one-year delay in seeking care, the use of light, controlled orthodontic forces allowed successful repositioning of extruded coronal fragments and preservation of pulp vitality. Conservative orthodontic management proved effective in this rare case of concurrent coronal extrusion and apical root fracture, supporting the healing potential of immature permanent teeth. Oral hygiene instructions and regular monitoring were essential components of care, reinforcing the importance of multidisciplinary, patient-centered approaches. This case contributes valuable evidence that, even in delayed presentations, careful planning and conservative treatment can result in favorable outcomes and preservation of the natural dentition in young patients. In conclusion, conservative management of extrusive luxation concurrent with root fracture in young permanent teeth may demonstrate a good prognosis in select cases, particularly when timely follow-up and proper oral care are maintained.

## Data Availability

The original contributions presented in this study are included in the article. Further inquiries can be directed to the corresponding author.
